# Galectin-3 in the Lateral Ventricle Regulates Immune Functions

**DOI:** 10.1080/17590914.2026.2622750

**Published:** 2026-02-07

**Authors:** Luana Campos Soares, Hana Bernhardova, Francis G. Szele

**Affiliations:** Department of Physiology, Anatomy, and Genetics, University of Oxford, Oxford, UK

## Abstract

Galectin-3 (Gal-3) is a protein expressed by glia that belongs to an ancient family. Gal-3 recognises molecular patterns on pathogens due to the high degree of its binding specificity with carbohydrate recognition domains. Thus, in sponges as well as other invertebrates, galectins are an important component of the primitive innate immune system. Whereas Gal-3′s function in driving mammalian inflammation is well known, its function in warding off bacterial and viral infections is not well appreciated. One route of brain infection is via the cerebrospinal fluid brain interface (CSFBI) which is primarily composed of ependymal cells (EC). ECs express high levels of Gal-3, and their motile cilia are compromised in Gal-3 KOs. In this mini-review, we discuss fundamentally important potential roles of Gal-3 in pathogen recognition at the CSFBI and suggest avenues of further study.

## Key Points

- Gal-3 binds to bacterial and viral glycoproteins.
- Gal-3 is selectively expressed in the ventricular-subventricular zone.
- Gal-3 may block pathogen entry into the brain via the cerebrospinal fluid brain interface.

## Introduction

Galectin-3 (Gal-3) is encoded by the LGALS3 gene and is composed of a carbohydrate recognition domain (CRD) and an N-terminal domain. The former binds glycosylated molecules and the latter causes multimerization of Gal-3 which then creates a molecular scaffold with myriad binding partners. These partners are often glycosylated receptors, such as BMPrα (Al-Dalahmah et al., 2020a), and the insulin receptor (Li et al., 2016). Gal-3 binding partners also include laminin, collagen, fibronectin, elastin, NG2 proteoglycans, integrins, NCAM, tenascin, and many other components of extracellular matrix signalling. Intriguingly, Gal-3 can also interact with many intracellular partners through protein-protein interactions (Dumic et al., 2006; Liu et al., 2002; Soares et al., 2021; Wang et al., 2004). This is the case for β-catenin, which is sequestered by Gal-3, causing down-regulation of Wnt signalling in postnatal ventricular-subventricular zone (V-SVZ) gliogenesis (Al-Dalahmah et al., 2020b).

Most of the literature shows that Gal-3 increases inflammation. Indeed, its previous names, Mac-2 and eBP, allude to its expression in macrophages and ability to bind to IgE antibodies. Gal-3 is expressed and secreted by immune cells such as microglia and has autocrine or paracrine effects (Li et al., 2016). For example, by cross-linking receptors including CEACAM3, FceRI and TLR4, Gal-3 activates monocytes/macrophages, microglia, neutrophils and mast cells (Burguillos et al., 2015; Feuk-Lagerstedt et al., 1999; Frigeri et al., 1993; Jeng et al., 1994; Liu et al., 1995; Yamaoka et al., 1995). Gal-3 is also a chemoattractant and increases monocyte and macrophage infiltration (Hsu et al., 2000; Li et al., 2016; Sano et al., 2000).

Gal-3 has been extensively studied in brain damage and disease, which usually increase its expression causing a variety of cellular responses. For example, Gal-3 activates microglia in a model of ischemic injury (Lalancette-Hébert et al., 2007). Also, in response to demyelination, increased levels of Gal-3 promote infiltration of leukocytes in the V-SVZ in a model of multiple sclerosis (James et al., 2016). Gal-3 expression levels are corelated with brain tumour grade and worse prognosis, not only due to its pro-inflammatory role, but also because it upregulates several pro-tumorigenic pathways (Bresalier et al., 1997; Camby et al., 2001; Gordower et al., 1999; Kuklinski et al., 2000; Wang et al., 2020).

## Galectin-3 Modifies Pathogen Infections

Galectins are important because of their ability to bind to pathogen glycoconjugates and also because they activate immune responses (Beatty et al., 2002), for example, during viral infections (Kuklinski et al., 2000; Machala et al., 2019; Sato & Nieminen, 2002; Wang et al., 2020). Galectins act on viral particles and infected cells by binding to glycosylated molecules such as viral envelope (ENV) proteins (Wang et al., 2020). Viral infections often increase Gal-3 expression, further intensifying inflammation (Machala et al., 2019; Wang et al., 2020). In mammals, Gal-3 regulates the innate and adaptive immune systems, and we hypothesise that it has important functions in COVID-19-induced damage. In another review, we discuss the potential role of Gal-3 in binding to and modulating the function of SARS-CoV-2 virus spike protein (Soares et al., 2021).

Gal-3 also directly interacts with and modulates bacterial infections (Table 1), reviewed in Díaz-Alvarez and Ortega (2017). Gal-3 can bind to bacterial lipopolysaccharide (LPS), via both its CRD and the N-terminal region (Kavanaugh et al., 2013; Mey et al., 1996). Other examples are Gal-3 CRD binding to the beta-galactoside-containing polysaccharide chain of *Klebsiella pneumoniae* and the N-terminal domain binding to *Salmonella minnesota* R7 LPS via the lipid A/inner core region of LPSs (Mey et al., 1996). Interestingly, Gal-3 can also direct antimicrobial guanylate binding proteins to vacuoles involved in bacterial secretion, thereby limiting bacterial activity (Feeley et al., 2017). Finally, Gal-3 has been shown to opsonise *Escherichia Coli* allowing microglial engulfment (Cockram et al., 2019). Thus, Gal-3 has distinct and important actions during bacterial infection.

**Table 1.** Galectin-3 binds to pathogens.

Gal-3 functions	Ligands	Pathogens	Effects	References
Pattern recognition receptor	LPS	*E. coli* *P. aeruginosa* *H. pylori* *N. gonorrhoeae*	Oligomerisation of Gal-3. Increased pro-inflammatory activity of Gal-3 on neutrophils. Negative regulation of LPS-mediated inflammation.	10.1371/journal.pone.0026004 10.1128/iai.65.7.2747-2753.1997 10.1046/j.1462-5822.2002.00219.x 10.1111/j.1462-5822.2005.00599.x 10.4049/jimmunol.181.4.2781
	LOS (Lipooligosacc-haride)	*N. gonorrhoeae*	Unclear – they showed Gal-3 binds LOS and that the target cells of gonococcal invasion constitutively express Gal-3.	10.1046/j.1462-5822.2002.00219.x
	LPG	*L. major*	Cleavage and truncation of Gal-3 retaining the C-terminal domain but lacking the N-terminal domain. The significance remains elusive.	10.1074/jbc.M201562200
	Oligomannans	*C. albicans*	Cross-linking of oligosaccharide components and Gal-3 binding directly induces fungicidal death in absence of any immune cells. Mechanism unknown but cross-linking of oligosaccharides in cell wall kills fungi.	10.4049/jimmunol.177.7.4718 10.1084/jem.20050749
	PI mannosides	*Mycobacterium* spp.	Gal-3 also accumulates in phagosomes containing live mycobacteria. Gal-3 was required for ubiquitin deposits on *M. tuberculosis* phagosomes. Gal-3 KO mice are more susceptible to *M. tuberculosis* infection but mechanism unknown.	10.1046/j.1462-5822.2002.00183.x 10.1016/j.devcel.2016.08.003
	Surface proteins 30, 32, 45, 64 70–80 kDa	*T. cruzi*	Gal-3 promotes adhesion of *T. cruzi* (primarily 70–80 kDa protein) to coronary artery smooth muscle cells and to extracellular matrix via a laminin bridge. *T. cruzi* cleaves Gal-3 and releases non-functional CTD. Blocking this cleavage leads to Gal-3 induced parasite death.	10.1016/S0014-5793(00)01347-8 10.1093/glycob/cwu103 10.1111/cei.13379
	Glycosyl & GlcNac	*S. pneumoniae*	Gal-3 increases susceptibility of influenza patients to *S. pneumoniae* infections by facilitating its adhesion to epithelia. Gal-3 increases neutrophil recruitment to the infected lungs and acts as a neutrophil-activating agent, increasing neutrophil phagocytosis of bacteria, and delaying neutrophil apoptosis. Gal-3 also bacteriostatic against *S. pneumoniae in vitro*.	10.1016/j.molimm.2014.12.010 10.4049/jimmunol.168.4.1813 10.2353/ajpath.2008.070870
Danger-associated molecular pattern	LPG *(L. major)* LPS *(N. meningitidis)* Oligomannans *C. albicans*	*L. major* *N. meningitidis* *C. albicans* *C. parapsilosis* *Franciscella* spp *H. pylori*	*L. major* – Gal-3 increased neutrophil recruitment in cutaneous leishmaniasis. *F. novicida* – Gal-3 induces leukocyte infiltration (mainly neutrophils), release of inflammatory cytokines and vascular injury. *N. meningitidis* – secreted Gal-3 increases adhesion to monocytes and macrophages. *C. albicans, C. parapsilosis* – treatment with exogenous Gal-3 increased phagocytosis of both *C. albicans* and *C. parapsilosis* and the exposure of neutrophils to *C. parapsilosis* yeast increased phagocytosis of *C. albicans* and was inhibited by anti-Gal-3 blocking antibody.	10.4049/jimmunol.1103197. 10.1371/journal.pone.0059616 10.1111/j.1462-5822.2012.01838.x 10.1111/cmi.12103
Immuno-modulator	LPS *Salmonella* spp Oligomannans *C. albicans* 30, 32, 45, 64 and 70–80 kDa surface proteins *T. Cruzi*	*M. leprae* *Salmonella* spp. *C. albicans* *T. cruzi* *T. Gondii* *S. cerevisiae*	*M. leprae* – Gal-3 increased IL10 release by monocytes. *Salmonella* spp – Gal-3 protects from LPS induced shock in by lowering secretion of pro-inflammatory cytokines and lowering NO production. *C. Albicans* – cytosolic Gal-3 that negatively regulates neutrophil ROS production and therefore prevents clearance of Candida infections. Contradictory role of extracellular Gal-3 which helps phagocytosis and kills *Candida. S. cerevisiae* and *C. albicans* – Gal-3 necessary for secretion of TNF-α by macrophages in response to *S. cerevisiae* and C. albicans. Gal-3 does not bind to *S. cerevisiae. T. cruzi* – Dendritic cells isolated from mice infected by T. cruzi showed reduced migration on Gal-3 coated surfaces. *T. Gondi –* Gal-3 induces pro-inflammatory response after infection and increased leukocyte infiltration into intestine, liver and brain. Gal-3 also induced monocytes/macrophages and CD8+ cell infiltration. However, dendritic cells isolated from Gal3^-/-^ mice secreted higher amounts of IL-12 which led to higher Th1-polarized response. Gal-3 therefore is proinflammatory and regulatory during *T. gondi* infection.	10.1093/infdis/jis920 10.4049/jimmunol.181.4.2781 10.3389/fimmu.2017.00048 10.1073/pnas.1111415108 10.1093/glycob/cwh068 10.2353/ajpath.2006.050636
Opsonin	Oligomannans *C. albicans* LPS *E. coli*	*Candida* spp. *E. coli*	Gal-3 participates in the recognition of *Candida* spp. by macrophages. *E. coli* – binding of Gal-3 increases phagocytosis by microglia.	10.4049/jimmunol.177.7.4679 10.3389/fimmu.2019.02647
Antimicrobial	Oligomannans (*Candida*) LPS *H. pylori*	*Candida* spp. *H. pylori*	*Candida* – binding and fungicidal activity proved, Gal-3 binding directly induces death in absence of any immune cells. Mechanism unknown but cross-linking of oligosaccharide components in the fungal cell wall is known to cause death. Gal-3 might induce this, but not confirmed. *H. pylori* – macrophages from Gal-3 deficient mice were inefficient in killing engulfed *H. pylori* in vitro. Extracellular recombinant Gal-3 has potent bactericidal effect on *H. pylori* (inhibited by lactose – suggesting Gal-3 CRD involved in this effect).	10.4049/jimmunol.177.7.4718 10.1084/jem.20050749 10.1111/j.14625822.2005.00599.x 10.1128/IAI.01299-15

Adapted with permission from Díaz-Alvarez and Ortega (2017).

Gal-3 can also affect vesicular membrane damage during infection. It is recruited upon the rupture of Shigella-containing phagosomes and adenovirus-containing endosomes (Maier et al., 2012; Paz et al., 2010). This is important for targeting the content of these vesicles for selective autophagy (Chen et al., 2014; Hong et al., 2019; Maejima et al., 2013). Recently, it was shown that Gal-3 associates with Alix, a component of the endosomal complex ESCRT and is recruited to sites of lysosomal damage by the transferrin receptor. There, it quickly promotes endomembrane repair (Jia et al., 2020). Gal-3 then switches interactions to associate with TRIM16, removing damaged lysosomes via autophagy (Chauhan et al., 2016; Jia et al., 2020). Gal-3 also promotes lysosome replacement via activation of TFEB (Jia et al., 2020). Interestingly, it has been recently reported that, in human macrophages, the Parkinson’s disease-related LRRK2 participates in the decision for membrane repair, while Gal-3 regulates destruction via autophagy (Herbst et al., 2020).

## Pathogens Entry into the Brain via the Lateral Ventricles

Ependymal cells (ECs) are multi-ciliated cells lining the ventricular system that form the CSFBI (Szele & Szuchet, 2003). EC’s are interconnected to one anotherdiscontinuous tight junctions but are also separated by the apical primary cilia of neural stem cells (NSC) which have direct contact with the CSF (Mirzadeh et al., 2008). This unique arrangement theoretically allows easier access from the CSF to the brain and could be a route of pathogen entry. The lateral ventricles encompass a large surface area that is bathed by CSF, produced by the choroid plexus (CP) (Dando et al., 2014). It has long been known that bacteria can accumulate in the CP and in the lateral ventricles in meningitis (Gilles et al., 1977). Similarly, *E. Coli* binds to CP cells, blood vessels and ECs (Parkkinen et al., 1988). Recently, a CSF virome has been described not just during infections but also in healthy people (Ghose et al., 2019).

**Figure 1.**
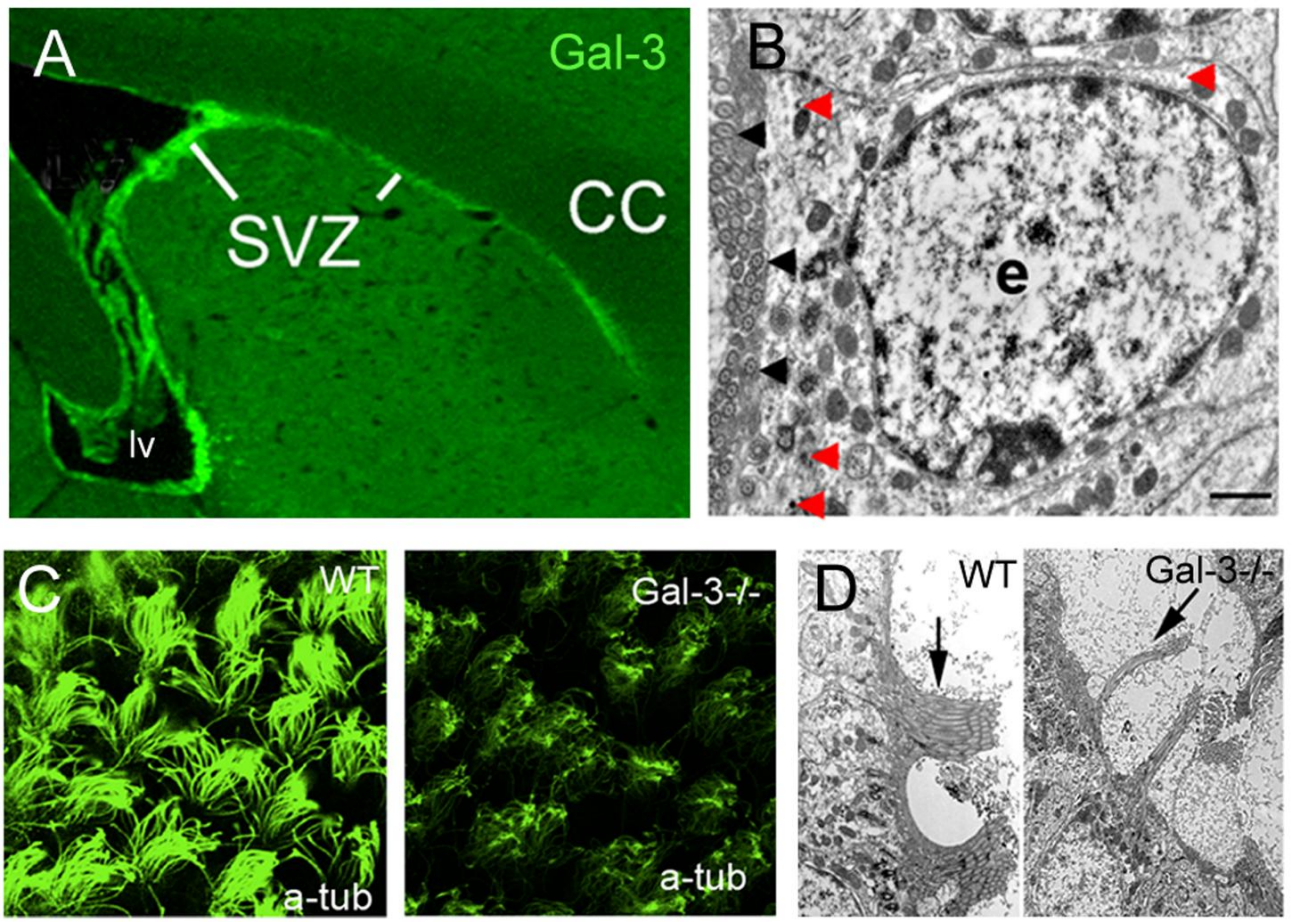
Galectin-3 functions in the wall of the lateral ventricle. (A) immunohistochemistry for Gal-3 in a WT adult mouse shows selective expression around the lateral ventricle (lv) and extending rostrally in the V-SVZ under the corpus callosum (cc). (B) Immuno-electron microscopy shows Gal-3 immunoprecipitates (ex. red arrows) in an ependymal cell (e). Note the microtubules at the apical (ventricular) surface of the cell (black arrowheads). Scale bar = 1 micron. (C) acetylated tubulin staining reveals healthy bundles of ependymal motile cilia in WT mice and massive disruption in Gal-3 knockouts. D) The same phenotype was observed with EM. Adapted with permission from Comte et al. (2011).

Gal-3 is usually expressed by microglia and macrophages during brain pathogenesis and was thought to distinguish infiltrating macrophages (Mac-2 high) from resting microglia (Mac-2 low). Thus, we were surprised to find that there is very little Gal-3 expression in microglia but robust expression in ECs and in the ventricular-subventricular zone (V-SVZ) stem cell niche which lines the basal side of ECs (Figure 1) (Comte et al., 2011). Microglia in the V-SVZ are constitutively semi-activated in healthy animals as evidenced by their morphology, marker expression and mitotic index (Goings et al., 2006). However, even in models of multiple sclerosis and stroke when there is a further activation of V-SVZ microglia, very few microglial cells in the V-SVZ expressed Gal-3 (Goings et al., 2008; Hillis et al., 2016; James et al., 2016; Young et al., 2014). These data suggest that the niche induces a unique pattern of Gal-3 expression, and that Gal-3 could regulate EC and NSC function.

**Figure 2.**
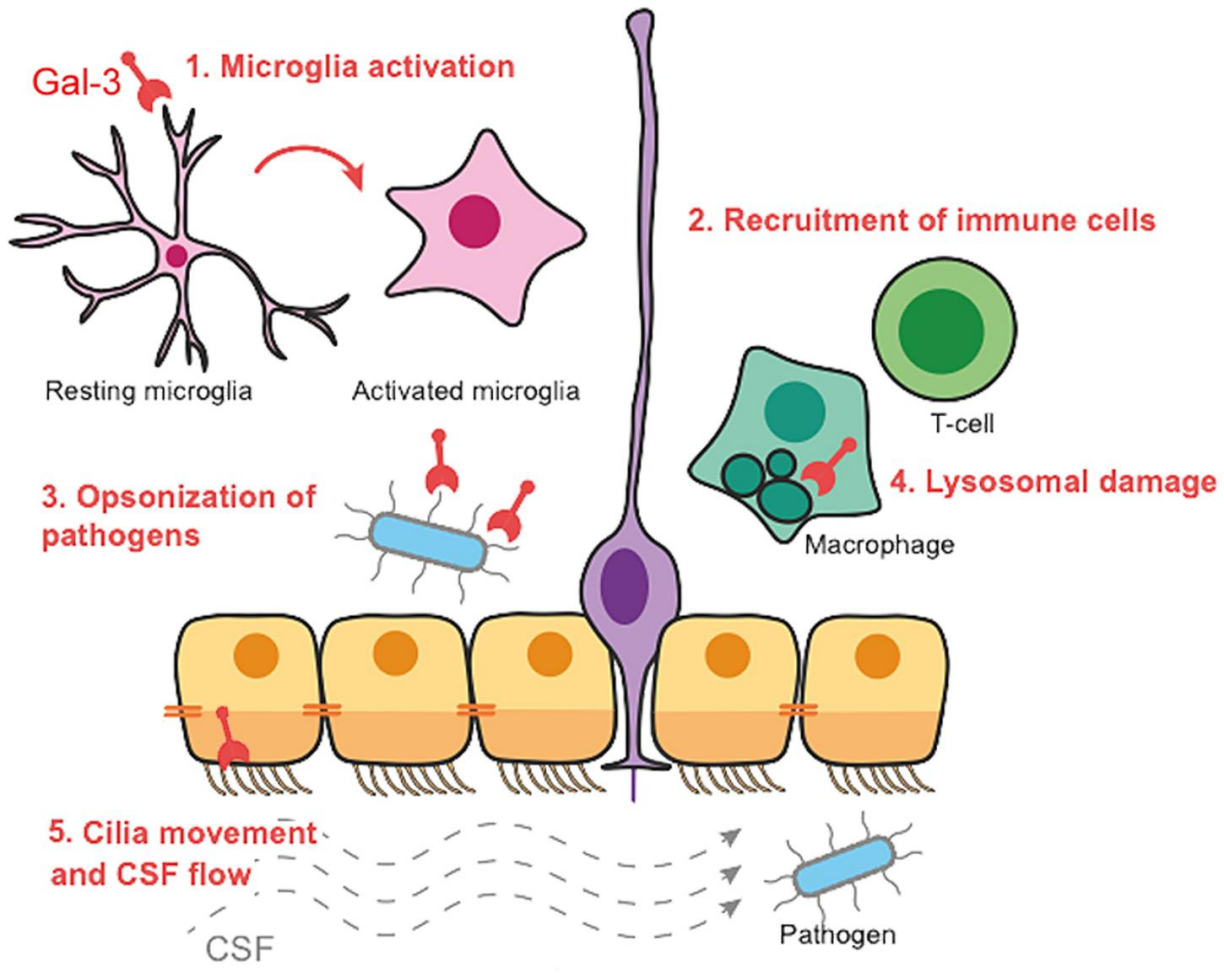
Schematic showing various potential functions of Gal-3 (red) in the V-SVZ stem cell niche.

Ependymal cell motile cilia exert the essential role of moving CSF through the ventricular system and thereby help expel pathogens from the brain. In our first study of Gal-3 KOs in the V-SVZ stem cell niche, we were struck by its profound role in motile cilia morphology (Comte et al., 2011). The knockout mice had fewer motile cilia, suggesting they were less efficient in propelling CSF through the ventricular system (Figure 1). In accordance, Gal-3 has been shown to affect motor cilia function in the respiratory tract (Clare et al., 2014). Gal-3 participates in the microtubule organisation of motile cilia, playing a role in cilia orientation, ciliary movement and consequently, fluid flow in the trachea (Clare et al., 2014). Taken together, it is likely that loss of Gal-3 could reduce EC ciliary motility and CSF movement and thereby regulate pathogen entry into the walls of the lateral ventricle. Based on studies from our group and others, Gal-3 is expressed at specific times and places in the brain and is thus positioned to influence specific aspects of disease and neural development. In the cells that form the CSF brain interface it has several integrated immunological functions that together could affect pathogen entry into the brain (Figure 2).

## Potential Roles of Galectin-3 in the Recognition of Pathogens at the CSF Brain Interface

Pathogens have been found in the CSF, in particular during meningitis. Real-time metagenomics has been useful in identifying viral pathogens in the CSF associated with meningitis (Morsli et al., 2022). Other work has also shown pathogen presence in the CSF including *Streptococcus pneumoniae*, *Neisseria meningitidis*, *Haemophilus influenzae*, *Staphylococcus*, and *E. coli* (Mengistu et al., 2013). These findings and compelling data from our group led to the hypothesis that Gal-3 behaves as an immunological barrier in the V-SVZ stem cell niche. This would indicate that the V-SVZ is not only neurogenic but also contributes to the cerebrospinal fluid brain interface (CSFBI) via Gal-3. The CP is highly vascularised and forms the blood-CSF barrier. Thus, the question arises to what extent do bacteria, viruses or other pathogens such as parasites or fungi cross the CP and enter the brain through the ependymal cell CSFBI.

Gal-3 expressed in the V-SVZ niche may also regulate inflammation and the immune reaction via control of chemokine expression and immune cell infiltration into the brain. As previously mentioned, Gal-3 is chemoattractant to macrophages in adipose tissue (Li et al., 2016) and future studies may show this role in the brain, inducing macrophages to enter the brain via ependymal cells and the V-SVZ. T cells enter the aging V-SVZ and diminish human and murine neurogenesis (Dulken et al., 2019; Moreno-Valladares et al., 2020). We showed that loss of Gal-3 blocked increases in levels of CCL2, CCL5, CCL8, and CXCL10 in the V-SVZ in a viral model of multiple sclerosis and consequently reduced T cell entry into the brain (James et al., 2016). Thus, multiple strands of data suggest that Gal-3 is expressed in cells lining the ventricles and has several immunological functions.

Our findings also imply the intriguing notion that neural cells in the V-SVZ may exhibit immune system functions and express immune markers. In support of this, we found that mimicking viral infection with PolyI:C in Dysbindin mutants (a model of schizophrenia) increases V-SVZ neural lineage cell expression of Tlr3, RelA and Sp1 – immune system molecules increased during inflammation (Al-Shammari et al., 2018). Similarly, the Ghosh group showed that continual expression of Tcf4 in adult hippocampal NSC is critically required to subvert a latent myeloid potential (Shariq et al., 2021). Deletion of Tcf4 in adult NSCs abrogated their neurogenic potential by transforming them into a myeloid-inflammatory state. Another study found evidence that V-SVZ neuroblasts have phagocytic functions (Lu et al., 2011). Thus, a new way of thinking about the neurogenic niches is that they have immunological roles and that the V-SVZ may participate in limiting infections spreading into the brain via the CSFBI.

Gal-3 in the V-SVZ may have several distinct functions that affect pathogen entry (Figure 2).

1. Pathogen-induced increases in Gal-3 expression could activate microglia and induce them to limit pathogen functions.
2. Gal-3 blockade could regulate macrophage and/or T cell V-SVZ infiltration, similar to what we showed previously (James et al., 2016) and these cells could also affect pathogen load in the V-SVZ and brain.
3. Gal-3 in the V-SVZ could also directly cause bacterial opsonisation (Kohatsu et al., 2006), the priming of antigens for phagocytosis, and thereby reduce their entry into the brain.
4. Gal-3 affects lysosomal function and autophagy in several systems (Chauhan et al., 2016; Herbst et al., 2020; Jia et al., 2020). Gal-3 could also affect lysosomes and autophagy in macrophages or microglia in the V-SVZ and thereby impact the ability to clear pathogens.
5. Ependymal cells (yellow/orange cells in Figure 2) are characterised by motile cilia which help move CSF through the ventricular system (Szele & Szuchet, 2003). Other work has shown that Gal-3 can affect motile cilia structure and function (Clare et al., 2014) and we showed that Gal-3 KO mice have stunted motile cilia. Thus we hypothesise that Gal-3 loss could decrease pathogen clearance through the ventricular system.

In some of these examples Gal-3 would have beneficial effects and in other cases detrimental. It is also likely that different pathogens would exert different effects. Thus, the balance of specific Gal-3 functions in pathogen entry will determine its targetability for clinical interventions.

## Conclusions

Thus, we suggest a major new role of V-SVZ cells and Gal-3 which is expressed in the niche. We believe they may function as an immunological barrier at the CSF brain interface to keep pathogen brain entry at bay. However, there are several open questions and important avenues for research. Definitive proof of our central hypothesis requires genetic removal or pharmacological blockade of Gal-3 to show this results in greater pathogen infiltration. It is also important to determine if pathogen entry into the V-SVZ increases Gal-3 expression. A more general question is if anti-inflammatories quell microglial activation in the V-SVZ and thereby increase the potential for brain infection? The exact mechanisms of V-SVZ cells and of Gal-3 in this new role may be revealed if Gal-3 mediated opsonisation occurs in the V-SVZ or if phagocytic and lysosomal properties are affected by Gal-3 in the V-SVZ. Finally, it will be important to determine the role of Gal-3 in motile cilia induced CSF movement and pathogen clearance. The answers to these questions, and others, will help clarify the role of the V-SVZ and Gal-3 in regulating pathogen entry through into the brain through the CSFBI.
